# Inflammatory Bowel Disease, Self‐Esteem and Sexual Well‐Being

**DOI:** 10.1155/nrp/4531024

**Published:** 2026-09-30

**Authors:** Kate O’Reilly, Eleanor Holroyd, Wenpeng You, Kath Peters

**Affiliations:** ^1^ Western Sydney University Locked Bag 1797 Penrith 2751 Australia westernsydney.edu.au; ^2^ Auckland University of Technology 55 Wellesley Street East Auckland 1010 New Zealand aut.ac.nz; ^3^ School of Nursing and Midwifery Western Sydney University Locked Bag 1797 Penrith 2751 Australia westernsydney.edu.au

**Keywords:** inflammatory bowel disease, self-esteem, sexual well-being, sexuality, women

## Abstract

**Aim:**

This research explored associations between self‐esteem, satisfaction with physical appearance, sexual satisfaction and physical and mental health among women living with inflammatory bowel disease in Australia.

**Introduction:**

Inflammatory bowel disease which includes Crohn’s disease and ulcerative colitis is a chronic autoimmune disease. Its relapsing, remitting nature has deleterious impacts on self‐esteem and sexual well‐being.

**Methods:**

The study employed a cross‐sectional design with secondary exploratory analyses and used an online survey to collect data from Australian women between March and November 2024.

**Results:**

Responses from 64 women revealed a nuanced but complex interrelationship between self‐esteem and biopsychosocial factors which impact on sexual well‐being. Self‐esteem scores for all women sat at the low end of normal, and none of the women reported any level higher than average. Women with lower self‐esteem reported more dissatisfaction with their physical appearance, poorer mental health and lower sexual satisfaction.

**Discussion:**

Having a high self‐esteem is a protective factor in adaptation to life events. The chronic nature of inflammatory bowel disease highlights that adaptation and self‐realignment are ongoing processes of which clinicians must be cognisant because chronic illness can have negative effects on self‐esteem.

**Conclusion:**

Sexual well‐being is influenced by multiple intersecting biopsychosocial factors. Routinely incorporating discussions which relate to mental health, self‐esteem and body image into assessments for people living with inflammatory bowel disease is critical in holistic care.

**Implications for Nursing:**

The findings provide justification for integrated teams of psychologists, sexual health nurses and potentially body‐image specialists which could be coordinated by registered nurses.

**Implications for Health Policy:**

Women‐specific care pathways which acknowledge reproductive life stages, relationship health, sexual well‐being, hormonal variation, menstrual impacts and sociocultural gender pressures are required. Introducing regular screening, which includes adding registries on digital platforms, may assist with ensuring equitable holistic healthcare.

## 1. Introduction

Inflammatory bowel disease (IBD) is classed as a chronic autoimmune disease which, as the name suggests, is characterised by an unregulated inflammatory response typically occurring in the gastrointestinal tract [1]. IBD most commonly includes Crohn’s disease (CD) and ulcerative colitis (UC), although other less common subtypes, such as microscopic colitis and IBD‐unclassified, are reported in the literature [2]. IBD may include gastrointestinal symptoms such as abdominal pain, fistula formation, faecal urgency and bloody diarrhoea. These, in addition to extraintestinal symptoms such as weight loss, debilitating fatigue, and joint pain, can have far‐reaching impacts on a person’s quality of life [3, 4]. While treatment options for IBD have and continue to improve, the relapsing‐remitting nature of IBD is destabilising, and recurrent flares of the disease can have deleterious effects on self‐esteem and sexual well‐being [5–8].

The prevalence of IBD globally is growing and is estimated to be 6.8 million people [9], according to IBD collaborators [10]. Within Australia, the prevalence of IBD was projected to reach 100,000 by 2022 [11]; however, more recent data indicate that this figure has nearly doubled, with a revised estimate of 179,423 people living with IBD [12]. The growth in IBD prevalence in Australia is exponential and is expected to be larger than growth in other similar high‐income countries such as New Zealand, the United Kingdom and Canada [12]. The GIBDC [10] identified that from the years 1990–2017, globally IBD was more prevalent in females (57%) than males (43%); however, diagnosis for women with IBD was often delayed due to diagnostic overshadowing with symptoms misattributed to menstruation or mental health concerns [12].

Delayed diagnosis due to dismissal of symptoms may have negative impacts on self‐esteem which is summarised as ‘a person’s evaluation of their worth as a person’ ([13] pp. 2). Crohn’s Colitis Australia & Insight Economics [13] reports that experiences that fall outside typical life patterns, such as illness, may decrease a person’s self‐esteem. This is important because the chronic relapsing‐remitting nature of IBD requires multifaceted responses to manage fluctuating symptoms. A cumulative negative impact on self‐esteem may result over the course of a person’s life as their contextual factors shift and change. Reitz [14] highlights the volume of self‐esteem literature published across various fields such as education and health and explains that high self‐esteem across the lifespan is important for adaptation and success in a range of areas such as relationships, work, and mental and physical health. Findings from the literature highlight that there is a bidirectional relationship between self‐esteem and all life domains, with sexual well‐being one of these [13–15].

The Chesli et al. [16] highlight that sexuality is socially constructed and, as with self‐esteem, is also influenced by the context in which a person lives. Sexuality is a core component of being human, and because of intersecting biological and environmental factors, it is experienced and expressed in individual ways. The literature highlights the negative impact on body image and sexual function following surgical and medical treatment for breast cancer [17], and while there is a growing body of work which is specific to IBD and sexuality, this remains limited within the Australian context. Von Hippel et al. [18] found that much of the research remains focused on sexual dysfunction and reproduction [19–22]. While this is an important contribution to clinical care for women with IBD, Von Hippel et al. [18] highlight that the broader concepts of sexuality are yet to be adequately considered. This study presents findings from broader mixed methods which incorporated a cross section of factors to more comprehensively explore the sexual and reproductive health needs of women who live with IBD in Australia.

### 1.1. The Study

#### 1.1.1. Aim

The specific aim of this study was to explore associations between self‐esteem, satisfaction with physical appearance, sexual satisfaction and physical and mental health among Australian women who live with IBD. Findings aim to inform an integrated medico‐social delivery model of healthcare which is inclusive of women’s overall well‐being.

## 2. Methods

### 2.1. Design

This study used a cross‐sectional design with secondary exploratory analyses and follows the STROBE checklist for reporting quantitative findings (Supporting File 1). A single survey comprising demographic questions and validated tools (following permissions) was developed by the first, second and fourth authors. The survey was then reviewed by two women who live with IBD to gain feedback about face and content validity and its suitability to fulfil the aim of the research. Feedback from experts with lived experience assisted with ensuring content validity. It was deemed that the included tools were relevant, and no adjustment to the wording was required. Advice regarding the ordering of sections within the survey was incorporated into revisions with the aim of ensuring logical progression for the participants completing the survey.

### 2.2. Study Setting and Recruitment

Inclusion criteria for this study were females over the age of 18 and living in Australia with a self‐reported medically confirmed diagnosis of IBD, either UC or CD. Participants were eligible to participate if they understood written and spoken English and were willing to complete an online survey which was estimated to take no longer than 30 min. The anonymous online survey was hosted on the Qualtrics platform between March and November 2024. National recruitment was conducted via Facebook and Twitter (now called *X*), as well as through online and print newsletters distributed by Crohn’s Colitis Australia and Ostomy Australia.

### 2.3. Sample Size

No formal a priori sample‐size calculation was undertaken because this was an exploratory study. Recruitment was conducted nationally during the predetermined data‐collection period from March to November 2024, and all respondents meeting the eligibility criteria during this period were included in the analysis. The resulting sample therefore represents a pragmatic sample determined by eligible participation within the available recruitment period and project resources.

### 2.4. Data Collection

The survey sought demographic information from respondents, which included age, diagnosis, education level, relationship status and living situation. Five self‐reported validated tools were used within the survey, which included a cross section of factors which collectively are considered important elements for sexual well‐being. Participants were informed that survey completion would take approximately 30 min. Participation was voluntary, and no incentives were offered. The SF‐12 survey [23] consists of 12 questions using both three‐ and five‐level Likert scales and is used to check a person’s general health and quality of life. Scoring is divided into the physical and mental components. A score of 50 represents the average of the population, with higher scores indicating higher than average levels of physical or mental health. The Women’s Health Questionnaire (adapted with permissions) [24] included eight questions related to sexual and reproductive health using a five‐point Likert scale ranging from *definitely* to *not at all* or *not applicable*. Findings from this section of the survey are presented as descriptive statistics. Scoring was not conducted, as the tool was not used in its entirety. The Sexual Satisfaction Scale (SSS), adapted from the Sexual History Form by the National Multiple Sclerosis Society [25], is comprised of four questions. Responses are recorded on a six‐point Likert scale, and scores can range from 4 to 24, with higher scores indicating higher levels of dissatisfaction. Sixteen of the 17 multiple‐choice questions from the Crohn’s and Colitis Pregnancy Knowledge Scale [26] were used. One which related to male fertility was removed, as the inclusion criteria for this study were women. A possible score between 0 and 16 could be recorded, with higher scores indicating a higher IBD pregnancy‐related knowledge. As the name suggests, the Rosenberg’s Self‐Esteem Scale (RSES) [27] measured women’s self‐esteem. Scoring of the 10 items used a four‐point Likert scale ranging from *strongly agree* to *strongly disagree*. Scores can range from 0 to 30, with lower scores indicating lower self‐esteem.

### 2.5. Data Analysis

After data from the 75 Qualtrics responses were uploaded into the Statistical Package for the Social Sciences (SPSS v29.0.1.0), 11 responses were removed because they did not meet the inclusion criteria of living in Australia, being female and/or having a diagnosis of IBD. Consequently, 64 records were included in the analysis. Descriptive statistics were used to summarise participant characteristics and study variables. Exploratory factor analysis using principal axis factoring was conducted to examine the dimensional structure of the 10‐item Rosenberg Self‐Esteem Scale (RSES) in this sample. Sampling adequacy was assessed using the Kaiser–Meyer–Olkin (KMO) measure, with values ≥ 0.60 considered acceptable, and Bartlett’s test of sphericity, with *p* < 0.05 indicating sufficient correlations for factor analysis [28]. Internal consistency was assessed using Cronbach’s alpha, with values ≥ 0.70 considered acceptable [28]. Factor retention was informed by eigenvalues > 1 and inspection of the scree plot [28, 29].

To address the study aim, Spearman rank correlations were used to examine associations between total RSES scores and satisfaction with physical appearance, sexual satisfaction and SF‐12 physical and mental component scores. Spearman correlation was selected because it is appropriate for examining associations involving ordinal and continuous variables and does not require normally distributed data. Continuous scale scores were retained rather than dichotomised. Pairwise complete cases were used for each analysis, with no imputation of missing data. All tests were two‐tailed, statistical significance was set at *p* < 0.05, and 95% confidence intervals were reported.

### 2.6. Ethical Considerations

Ethics was granted by Western Sydney University (Approval Number: H15913) to share the anonymous survey online using the Qualtrics platform. Prior to completion of the anonymous survey women were required to read a participant information sheet which outlined the study aim. Women ticked a consent box to indicate they understood the purpose of the study and to confirm that they understood that completion of the anonymous survey implied consent. A list of counselling and support services was included at the end of the survey to ameliorate a potential risk that completing the survey may cause some emotional distress for some participants.

## 3. Results

### 3.1. Participant Characteristics

Survey respondents ranged in age from 19 to 59 years (mean = 36 years). Most participants lived in New South Wales/Australian Capital Territory (34.4%; *n* = 22) or Victoria (28.1%; *n* = 18). Most participants reported a diagnosis of CD (73.4%; *n* = 47), with the remainder reporting UC (26.6%; *n* = 17). Most were in paid employment (78.1%; *n* = 50), and over half (54.7%; *n* = 35) reported earning more than AU$60,000 annually. Participants who were not in paid employment were students, carers, or receiving a government‐assisted pension because they were too unwell to work due to their IBD. Most participants were married or in de facto relationships (68.8%; *n* = 44).

### 3.2. Sexual Well‐Being, Physical Appearance and Health Outcomes

Physical health outcomes were suboptimal, with 67.0% (*n* = 63) of participants scoring below 50 on the SF‐12 Physical Component Summary (PCS) [23]. Mental health outcomes were also below the population benchmark, with 81.0% (*n* = 63) scoring below 50 on the SF‐12 Mental Component Summary (MCS).

Menstrual symptoms were commonly reported, with 72.4% (*n* = 58) experiencing abdominal cramps during the menstrual cycle, 51.7% (*n* = 58) reporting breast tenderness and 44.8% (*n* = 58) reporting heavy periods. Over half of participants (53.1%; *n* = 64) reported changes in their menstrual cycle following their IBD diagnosis. Cervical cancer screening within the previous 5 years was reported by 73.4% (*n* = 64) of participants, while 50.0% (*n* = 64) reported performing monthly breast self‐examinations.

A majority of respondents (89.4%; *n* = 47) reported noticeable physical changes in their bodies following diagnosis of IBD. Among the 59 participants who responded to the physical appearance satisfaction item, 16.9% (*n* = 10) reported being satisfied, 33.9% (*n* = 20) were neutral and 49.2% (*n* = 29) reported being dissatisfied. The SSS was completed by 56 participants, with a mean score of 12.39 (SD = 6.29); higher scores indicated greater sexual dissatisfaction.

### 3.3. Self‐Esteem and RSES Factor Analysis

Fifty‐nine participants completed the RSES. The mean self‐esteem score was 15.75 (SD = 1.54), which was at the lower end of the reported normal range of 15–24 [27]. The KMO value of 0.873 exceeded the recommended minimum of 0.60, and Bartlett’s test of sphericity was significant (*χ* ^2^ = 355.696, df = 45, *p* < 0.001), supporting the suitability of the RSES item correlations for factor analysis [28]. The internal consistency of the RSES was excellent, with a Cronbach’s alpha of 0.916 for its 10 items, confirming the reliability of the scale and the appropriateness of the data for factor analysis [28]. Table 1 highlights that one factor had an eigenvalue greater than 1, meeting the criterion for factor retention. This finding was further corroborated by the scree plot (see Figure 1), which displays eigenvalues in descending order. The plot clearly shows a single eigenvalue exceeding 1 before reaching the ‘elbow’ point, affirming the unidimensionality of the scale and the robustness of the factor solution.

**TABLE 1:** Total self‐esteem variance explained.

Factor	Initial eigenvalues			Extraction sums of squared loadings		
	Total	% of variance	Cumulative (%)	Total	% of variance	Cumulative (%)
1	5.788	57.878	57.878	5.358	53.581	53.581
2	0.913	9.127	67.004			
3	0.788	7.884	74.889			
4	0.732	7.320	82.209			
5	0.446	4.464	86.673			
6	0.405	4.047	90.720			
7	0.336	3.363	94.082			
8	0.225	2.248	96.330			
9	0.219	2.185	98.516			
10	0.148	1.484	100.000			

*Note:* Extraction method: principal axis factoring.

**FIGURE 1:**
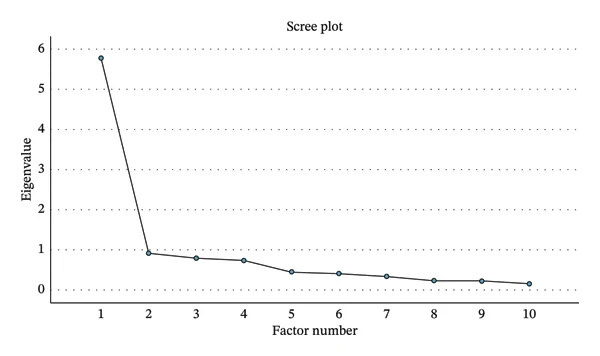
Scree plot.

Exploratory factor analysis using principal axis factoring indicated that all RSES [27] items loaded onto a single factor, confirming the scale’s unidimensional structure in this sample (see Table 2). The five‐iteration extraction process supported the interpretation of a single underlying construct representing global self‐esteem.

**TABLE 2:** Item‐level factor loadings for the global self‐esteem factor.

Factor 1 (unidimensional): global self‐esteem (numbered as per Rosenberg Self‐Esteem Scale)	Factor loading (correlation coefficients)
10) I take a positive attitude toward myself	0.877
9) All in all, I am inclined to feel that I am a failure	0.832
3) I feel that I have a number of good qualities	0.781
6) I certainly feel useless at times	0.796
2) At times, I think I am no good at all	0.755
7) I feel that I’m a person of worth, at least on an equal plane with others	0.730
5) I feel I do not have much to be proud of	0.685
8) I wish I could have more respect for myself	0.641
1) On the whole, I am satisfied with myself	0.631
4) I am able to do things as well as most other people	0.523

### 3.4. Associations Between Self‐Esteem and Study Outcomes

Spearman rank correlations showed that higher self‐esteem was significantly associated with greater satisfaction with physical appearance (*ρ* = −0.385, *n* = 59, *p* = 0.003, 95% CI [−0.589, −0.136]) and greater sexual satisfaction (*ρ* = −0.355, *n* = 51, *p* = 0.010, 95% CI [−0.580, −0.080]) (Table 3). Higher self‐esteem was also significantly associated with better mental health (*ρ* = 0.308, *n* = 58, *p* = 0.018, 95% CI [0.047, 0.531]). The association between self‐esteem and physical health was positive but not statistically significant (*ρ* = 0.225, *n* = 58, *p* = 0.089, 95% CI [−0.043, 0.463]).

**TABLE 3:** Associations between self‐esteem and study outcomes.

Outcome	*n*	Spearman’s *p*	95% CI	*p*
Satisfaction with physical appearance	59	−0.385	−0.589 to −0.136	0.003
Sexual satisfaction	51	−0.355	−0.580 to −0.080	0.010
SF‐12 Physical Component Summary	58	0.225	−0.043 to 0.463	0.089
SF‐12 Mental Component Summary	58	0.308	0.047 to 0.531	0.018

*Note:* Higher scores for physical appearance satisfaction and the Sexual Satisfaction Scale indicate greater dissatisfaction; therefore, negative correlations indicate that higher self‐esteem was associated with greater satisfaction.

## 4. Discussion

This study aimed to examine associations between self‐esteem, satisfaction with physical appearance, sexual satisfaction and physical and mental health among women living with IBD in Australia. The findings presented in this study highlight the nuanced but complex interrelationship between self‐esteem and various biopsychosocial factors which can impact on sexual well‐being. While the mean RSES [27] score of 15.7 reported by women in this study sits within the normal range of self‐esteem, it was on the very lowest end of the range, and none of the women reported any level higher than average. This is an important finding because Reitz [14] explains that high self‐esteem is a protective factor in adaptation to life events. O’Reilly et al. [30] highlight there is an abundance of in‐depth qualitative literature which pertains to resilience in chronic conditions and trauma, suggesting that only people who are strong will overcome the impact on their daily lives. Of importance is that the social construct of normalcy underpins this notion, subtly suggesting that if people do not ‘bounce back’, they are burdensome, less able and not coping because they have not adapted in a way that is valued by society [31]. O’Reilly et al. [30] challenge the accurateness of the term resilience explaining that the term tenacity more suitably reflects the persistence required to live with lifelong impairments, which in the case of IBD, may unexpectedly reoccur [1]. The chronic nature of IBD and the range of impacts reported previously highlight that for many people, adaptation and self‐realignment are ongoing processes of which clinicians must be cognisant because illness can have negative effects on self‐esteem [13]. This is relevant to highlight because more than half (50.6%) of the estimated 180,000 Australians who live with IBD are experiencing active disease [12].

Our study shows that women reporting a lower self‐esteem who live with IBD, unsurprisingly, have more dissatisfaction with physical appearance. Women in this study with average scores of self‐esteem did report more satisfaction with their physical appearance, although they still reported changes in their bodies due to IBD. Our research did not specifically measure body image, although we asked participants questions about their perceived changes in physical appearance. This is important in relation to sexual well‐being because Elias et al. [32] note that initiating or being receptive to sexual activity can be influenced by self‐perceptions of one’s body, which is influenced by sociocultural factors and remains fluid across the lifespan [33]. This highlights that context is important and ever‐changing, as body image is responsive and is shaped by the fluctuating and visible impacts of IBD. This is particularly relevant for women who live with IBD because following diagnosis they may deviate from the societal popularised norms of bodily perfection which continue to be widely perpetuated [34]. Paudel et al. [33] explain that popularised ideals of beauty may be used as personal reference points to compare oneself against. If a person perceives they do not meet the socially constructed beauty standard, this negatively impacts their feelings of self‐worth, which is a core element of self‐esteem [14]. Some bodily deviations due to IBD are reported by O’Reilly et al. [9] along with Fourie et al. [6], who highlight that women’s changing bodies may be due to faecal incontinence and urgency, which may fluctuate and become more difficult to manage throughout menstrual cycles. Additionally, the aforementioned authors highlight that women reported invasive surgical procedures which may result in scarring, the insertion of seton drains to manage fistulas or stoma formation following colorectal resection. Fluctuating weight due to medications and active disease, facial hair and development of skin tags close to genitalia are all examples of deviations from popular cultural images of beautiful bodies which can negatively impact on self‐esteem and sexual well‐being [32, 35].

Most women in this study who completed the SF‐12 tool [23] within the survey reported lower than average physical health (67.0%; *n* = 63) and less than average mental health (81.0%; *n* = 63), which is concerning. Elias et al. [32] note that mood disturbances such as anxiety and depression are commonly reported in people who live with IBD. Findings from our study align with Weiss et al. [36], reporting that more frequent occurrence of physical symptoms related to illness increases the likelihood of depressive symptoms and may also reflect that more of the women who responded to the survey may have had active disease [37]. While the range of self‐esteem scores in this study was narrow, our analysis showed that lower self‐esteem was associated with greater dissatisfaction with physical appearance, lower mental health and lower sexual satisfaction; the association with physical health was positive but not statistically significant. The intersection of all of these factors and their impact on sexual well‐being is not surprising; however, assessment of self‐esteem for a person who lives with IBD is not commonly undertaken during a routine clinic review. Interventions aimed at assessing and enhancing self‐esteem may have significant psychosocial benefits for individuals managing chronic conditions such as IBD.

### 4.1. Implications for Nursing and Health Policy

These findings highlight the need for gender‐sensitised healthcare delivery. The incorporation of psychosocial screening for self‐esteem, body image and sexual well‐being for women who live with chronic conditions could identify those at risk of emotional distress. The findings provide empirical justification for integrated teams of psychologists, sexual health nurses and potentially body‐image specialists which could be co‐ordinated by registered nurses. Also important is the development of women‐specific care pathways acknowledging reproductive life stages, relationship health, sexual well‐being, hormonal variation, menstrual impacts and sociocultural gender pressures. Introducing regular psychosocial screening, which includes adding digital registries, may assist with ensuring equitable holistic healthcare, as patterns of delays and underscreening may become more visible. Digital platforms can provide clinicians with reminders to ensure systematic identification of needs and may help to improve continuity of care by enabling shared documentation and communication across integrated multidisciplinary teams. Digital registries embedded into digital platforms may be helpful in shifting from reactive, episodic models of care to a truly person centred approach.

### 4.2. Limitations

Some limitations of this study include the small sample size and variability in responses across sections of the survey. The geographic distribution of participants, with a large proportion living in New South Wales, together with online recruitment, may have favoured more health‐engaged participants with greater access to specialist healthcare. While online recruitment can extend reach across a geographically dispersed population such as Australia, it assumes reliable internet access and sufficient digital literacy. This limits the extent to which the findings can be generalised to women living in rural and remote areas, and the results should therefore be interpreted with caution.

Given that few older women participated, the study is unable to fully represent how ageing, longer disease duration and changing social roles and health priorities may shape women’s needs across the lifespan. Disease activity, disease severity and remission status were not collected; therefore, participants’ accounts cannot be specifically situated in relation to active disease. However, the study meets the criteria for an exploratory pilot investigation and provides valuable preliminary insights to inform future research. Numerous broad‐based attempts were made to increase survey participation; however, funding constraints determined the data collection timeline.

The modest sample size may have reduced the precision of the estimated associations and limits the generalisability of the findings to the broader population of women living with IBD in Australia. Accordingly, the observed associations should be interpreted as exploratory and require confirmation in larger, adequately powered studies. Due to the chronic, relapsing‐remitting nature of IBD, longitudinal research exploring self‐esteem in this population may provide greater insights.

### 4.3. Recommendations for Further Research

Due to the chronic, relapsing remitting nature of IBD, a longitudinal study which explores self‐esteem in this population may provide greater insights to inform care delivery.

## 5. Conclusion

Sexual well‐being is a broad concept which is influenced by multiple intersecting biopsychosocial factors. Routinely incorporating discussions which relate to mental health, self‐esteem and body image into assessments for people living with IBD is an important aspect of a holistic approach to address sexual well‐being.

## Funding

Early Career Fellowship grant funding from Western Sydney University bought out teaching time for the first author to conduct the research.

Open access publishing facilitated by Western Sydney University, as part of the Wiley ‐ Western Sydney University agreement via the Council of Australasian University Librarians.

## Conflicts of Interest

The authors declare no conflicts of interest.

## Supporting Information

Additional supporting information can be found online in the Supporting Information section.

## Supporting information


**Supporting Information** STROBE statement—checklist of items that should be included in reports of observational studies.

## Data Availability

The data that support the findings of this study are available on request from the corresponding author. The data are not publicly available due to privacy or ethical restrictions.
